# Using Behavioral Skill Training to Extend Discrete-Trial Teaching Skills Across Instructional Programs

**DOI:** 10.1007/s40617-025-01094-5

**Published:** 2025-09-25

**Authors:** Heidi Skorge Olaff, Ida Bredahl Koch, Denys Brand

**Affiliations:** 1 Faculty of Health Science, Institute of Behavioural Science, OsloMet––Oslo Metropolitan University, Post Box 4, St. Olavs Plass, 0130 Oslo, Norway; 2 Oslo Community, Oslo, Norway; 3 California State University––Sacramento, Sacramento, CA USA

**Keywords:** Behavioral skills training, Discrete-trial teaching, Generality, Social validity

## Abstract

Educators serve an essential role when teaching skills to children with special needs, and effective training is crucial. Behavioral skills training (BST) is an empirically supported training approach that has been used to establish discrete trial teaching (DTT) skills in school staff. This study replicates previous research by assessing the effect of BST when teaching DTT skills and their extension across instructional programs and settings not associated with training. Results showed that BST resulted in the extension of DTT skills across instructional programs that were maintained three weeks after training was completed. BST was rated favorably in a social validity survey.

**Supplementary Information:**

The online version contains supplementary material available at https://doi.org/10.1007/s40617-025-01094-5.

Given the essential role that educators play in the education of children with disabilities and the cost associated with these services, it is essential to provide teachers with effective, evidence-based training. Behavioral skill training (BST) is an empirically supported training package consisting of instructions, modeling, rehearsal, and feedback and has been used to teach a variety of functional skills (e.g., Parsons et al., 2013), including the application of discrete-trial teaching (DTT; Smith, 2001) that often requires extensive staff training.

DTT is a frequently used procedure for teaching functional skills to children with disabilities. An important and often overlooked objective when training staff to implement DTT is for newly acquired DTT skills to extend across instructional programs not directly taught during training (Briggs et al., 2024; Frank-Crawford et al., 2024). The extension of DTT skills across instructional programs may result in labor savings as staff do not have to be retrained every time they encounter novel instructional programs. Assessing the extension or generality of DTT skills across instructional programs does not appear to be common practice. In their review, Briggs et al. (2024) reported that only 74% of studies that examined the generality of DTT skills assessed generality across novel instructional programs. Moreover, few studies have examined the social validity of BST. In a recent review of staff training procedures (including BST), Hickey et al. (2024) found that only 46.7% of studies reported social validity data. Measuring social validity is essential to ensure the appropriateness of behavior change, acceptability of procedures, and meaningfulness of the target behavior (Huntington et al., 2023).

Although previous research has investigated the use of BST to achieve generality of DTT skills with staff, there are relatively few studies measuring the extension of DTT skills following BST across different instructional programs. For example, Sarokoff and Sturmey (2008) assessed stimulus generality across instructional programs, focusing on listener behaviors (e.g., identification of body parts and gross motor imitation) with staff in a school for children with autism spectrum disorder (ASD). Results demonstrated that BST produced generalized DTT skills to novel listener targets and students. The present study extends Sarokoff and Sturmey (2008) by assessing the extension and maintenance of DTT skills across different academic instructional programs rather than evaluating stimulus generalization across listener programs that share some relevant properties. The primary aim of this study was to use BST to establish the correct implementation of DTT skills with school staff who provide educational services to students with ASD. The secondary aims were to evaluate whether DTT skills extended across academic instructional programs, extended to a setting that differed from treatment, and maintenance of established DTT skills. Finally, we examined the social validity of using BST to train DTT skills.

## Method

### Participants, Setting, and Materials

Three teaching assistants (one self-identified as a woman and two as men), aged 20–30, participated. One participant had previous experience with DTT. The three teaching assistants worked with the same student, a 7-year-old boy diagnosed with ASD with specific language and comprehension difficulties. The study was conducted during the COVID-19 pandemic, and, therefore, one student was involved in the study to minimize infection risk. All procedures were conducted in the student’s classroom (located in the special education department of a primary school in Norway).

Materials consisted of a math task (10-friends) with number cards. An example of 10-friends is that 8 is the “friend” of 2 because their sum is 10. For the five extension tests, materials included (1) images of objects and cards with written words in lowercase letters (reading comprehension), (2) cards with numbers from 1–20 and cards with images of coin quantities (matching numbers with correct quantity of coins), (3) cards with general questions (vocally answering general questions), (4) a poster with pictures of analog clocks and corresponding cards with digital times (matching digital to analog times), and (5) cards with numbers from 1–20 and cards with images of marker quantities (matching numbers with quantity of markers). Finally, during the setting-extension test, we used a bingo game. A previously established token economy was used during all instructional programs. When the student earned 12 tokens, he could exchange them for a fictional key to open a treasure box that contained 10 different notes with attractive surprises. A video camera was used to record all sessions.

### Response Measurement

The primary dependent variable was the percentage of correct implementation of 10 DTT skills by the participants during 5-min sessions (Koegel et al., 1977). Correct implementation of a DTT skill was recorded as plus ( +), incorrect/no response was scored as minus ( −), or N/A (not applicable). See Appendix A. The percentage of correct implementation was calculated by dividing the number of correctly implemented DTT skills by the total number of DTT skills and multiplying the resulting ratio by 100. Participants were provided with a prepared scoring sheet to score student responses to each 10-friends trial. The mastery criterion during BST training tests was three consecutive 5-min sessions with at least 90% correct implementation of DTT skills. For the across instructional programs-extension tests, the mastery criterion was three consecutive 5-min sessions with at least 90% correct implementation of DTT skills across three different instructional programs. During the setting-extension test, post, and maintenance tests, the mastery criterion was at least 90% correct implementation of DTT skills in one 5-min session.

#### Interobserver Agreement

Two independent observers collected data from videos for at least 30% of sessions for each participant for reliability purposes. We calculated interobserver agreement (IOA) by using point-to-point agreement in 30-s intervals. IOA scores were calculated by dividing the total number of agreements by the total number of agreements and disagreements and converting the ratios into an overall percentage for each 5-min session. Mean IOA for correct DTT implementation was 94% across the 10 DTT components (range 87.7–100%).

#### Procedural Fidelity

Procedural fidelity (PF) data were collected via video recordings by an independent observer for at least 30% of BST sessions across participants. The observer scored whether the experimenter (a) provided written and verbal instructions for each of the 10 DTT skills, (b) initiated each session by having the participants watch the video model at least three times, (c) gave the participants at least three training trial practice opportunities, and (d) provided at least three instances of positive feedback for every corrective feedback statement. Scores were calculated by dividing the number of correctly performed BST steps by the total number of BST steps and converting the resulting ratio into a percentage. Mean PF was 95% across all BST steps (range 88–100%).

### Procedure

A concurrent multiple-baseline design across participants was used to evaluate the effects of BST on each participant’s correct implementation of DTT skills.

#### Baseline Tests

Participants were informed that they would be asked to teach 10-friends to the student using DTT. Similar to Sarokoff and Sturmey (2008), the experimenter provided vocal and written instructions for DTT prior to the first baseline test. Providing instructions before the first baseline test helped to ensure that participants knew what was expected of them and permitted examination of the effects of instructions alone on correct DTT implementation. During baseline tests, participants were provided with task materials, scoring sheets for the 10-friends trials to be conducted in the 5-min sessions, and the student’s token economy board. Participants were required to record correct, incorrect, and prompted responses emitted by the student during each session. Before each baseline test, the participants were given an instruction to conduct a DTT (“Implement a training session using DTT with 10-friends for 5 min as best you can”). The experimenter then started a timer counting down from 5 min.

#### Training Tests

Before each BST session, a training test was conducted. Training tests were identical to baseline tests except that the experimenter did not provide vocal and written instructions for DTT to the participants. Before training tests, the experimenter instructed participants to “Implement a training session using DTT with 10-friends for 5 min as best you can.” The experimenter then started a timer counting down from 5 min. After each training test, a BST session commenced.

#### Behavioral Skills Training

**Instructions.** Before the first baseline test, the experimenter provided vocal and written instructions on DTT.

**Modeling.** Participants watched 24-s video clips of a person correctly implementing DTT to teach 10 friends. The video clip consisted of one 10-friends trial and was repeated three times.

**Rehearsal.** Participants engaged in role-play to practice DTT skills with the experimenter playing the role of the learner. During role-play, participants rehearsed the implementation of all 10 DTT skills in the context of teaching 10 friends, including the use of a progressive prompt delay procedure. The criterion for increasing the prompt delay was a correct response on two consecutive trials. If an incorrect response occurred on one trial, the prompt delay was shortened to the previous level, for example, from 4 to 3 s.

**Feedback.** The experimenter provided feedback to participants during role-plays on their correct and incorrect implementation of DTT skills. Positive feedback consisted of enthusiastic, behavior-specific praise and comments (e.g., “You did a good job reinforcing the student’s correct response, you reinforced immediately and had an enthusiastic tone of voice”). The researcher provided at least three positive feedback comments for each corrective feedback statement. Corrective feedback consisted of comments about incorrect implementation (e.g., “The delivery of the consequence was too slow; try to deliver the consequence more quickly next time”).

#### Extension Tests Across Instructional Programs and to a Setting Not Included during Training

At least three extension tests across instructional programs were conducted in a random sequence. Tests for the extension of DTT across instructional programs consisted of novel programs (i.e., reading comprehension, counting quantities and matching with the correct number, and answering general information questions) that were not targeted during BST. When the participants correctly implemented DTT skills in the presence of unfamiliar antecedents and unknown responses (from the student), we scored it as DTT skills having extended across that particular instructional program. If the correct implementation of DTT skills was not observed during the first extension test for a given instructional program, BST sessions were conducted to teach the participant to correctly implement DTT on the relevant instructional program. After the BST mastery criterion was met for the targeted instructional program, tests for the extension of DTT skills across the remaining instructional programs resumed. Finally, after the mastery criterion of the DTT-extensions tests was met, a test for the extension of DTT skills in a setting that differed from the training setting was conducted in a common area at school and consisted of the participants using DTT skills to teach the student a bingo game with a peer. Before each extension test, the same instruction was given as during baseline and training tests.

### Post-Test and Maintenance Test

All participants underwent a post-test following the setting-extension test, and a maintenance test was completed three weeks later. Post- and maintenance tests were identical to the baseline tests (10-friends). Before post- and maintenance tests, the participants were given the same instruction as during baseline and training tests: The participants were simply asked to conduct a DTT session for 5 min. As during the former tests, the experimenter recorded the presence or absence of the 10 DTT components. Before rehearsal, the same instruction as during baseline tests were given (“Implement a training session using DTT with 10-friends for 5 min as best you can”).

### Social Validity

Following the study, participants completed a 6-item social validity survey, which was administered non-anonymously and consisted of a Likert-like scale ranging from 1 (strongly disagree) to 5 (strongly agree), as well as two multiple-choice questions (Table 1).

**Table 1:** Results from the social validity survey

Questions	P1	P2	P3	Mean
1. *Rate from 1–5 how you liked the different components of BST*				
(a) Instruction	4	3	4	3.66
(b) Modeling	3	4	2	3
c) Rehearsal (role play)	2	2	3	2.33
d) Feedback	5	5	5	5
*2. Rate from 1–5 how effective you consider each component of the BST-supervision*				
(a) Instruction	5	4	4	4.33
(b) Modeling	2	2	2	2
(c) Rehearsal (role-play)	4	2	4	3.33
(d) Feedback	4	5	5	4.66
*3. Which component was for you the most effective during supervision to master effective delivering of reinforcers?*				
Answers from the participants	Rehearsal	Instruction	Feedback	
*4. Which of the components did you like the most?*				
Answers from the participants	Rehearsal	Feedback	Feedback	
*5. Have you noticed an improvement in your abilities to effectively deliver reinforcers after the supervision compared to before?*				
	5	5	4	4.66
*6. Do you feel more competent related to teaching the learner now than before the supervision?*				
	5	3	4	4

*P* Participant.

## Results and Discussion

**Fig. 1:**
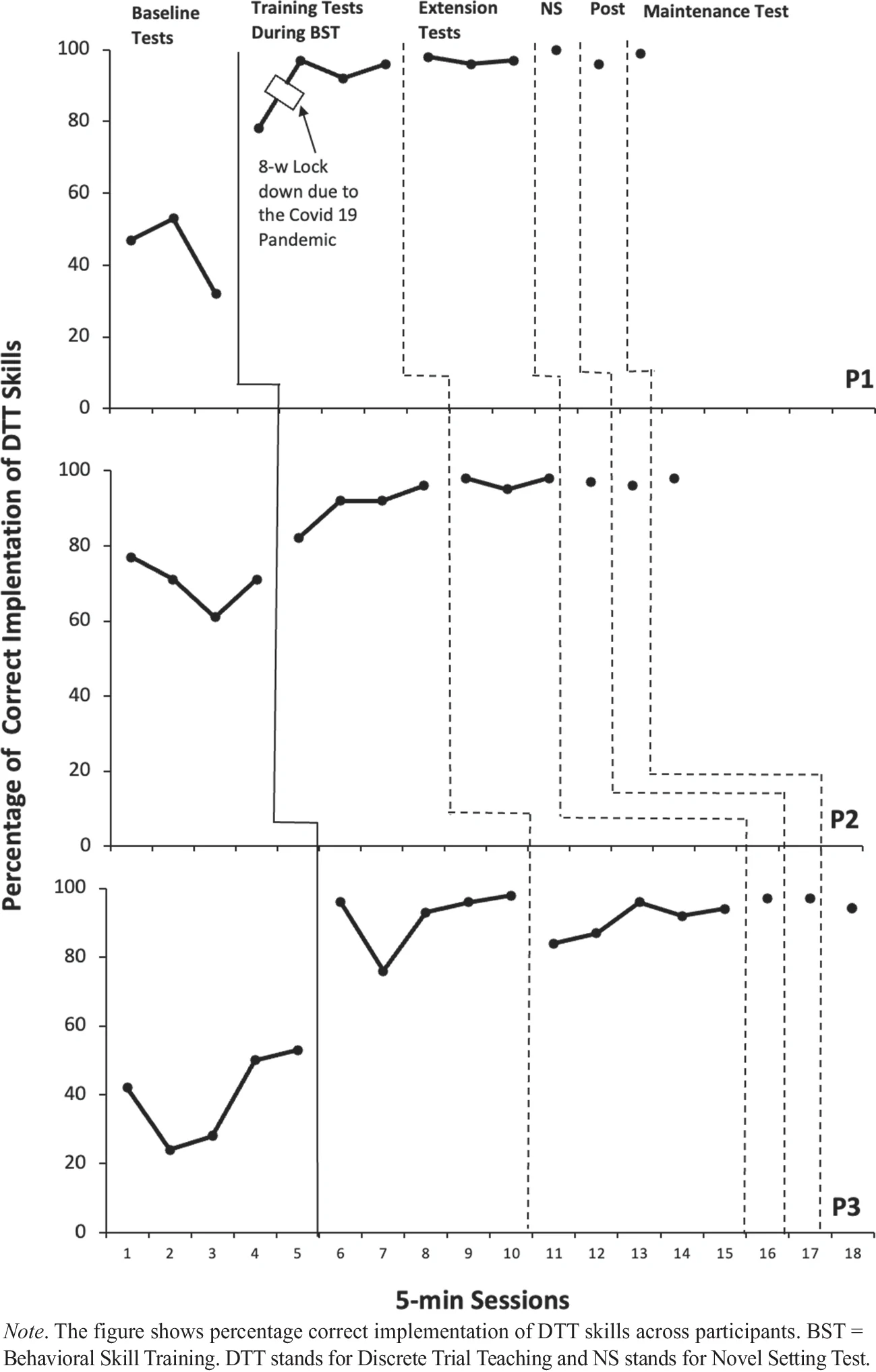
The effect of behavioral skill training on discrete trial teaching skills

The purposes of the present study were to train educators using BST to develop DTT skills, to evaluate the extension and maintenance of newly acquired DTT skills across instructional programs when teaching a student with ASD, and to assess the social validity of BST in training DTT skills. Figure 1 depicts the percentage of correct DTT skills implemented by each participant across all tests.

To meet the mastery criterion for DTT skills during BST training tests, four (P1 and P2) to seven (P3) BST sessions were required. The mean percentage of correct implementation for P1 increased from 44% during baseline tests (range 32–52%) to 91% during BST training tests (range 78–97%). During the extension tests, P1 achieved a mean correct implementation score of 98% (range 96–98%) across three tests and 100% correct implementation during the setting-extension test. P2 increased from a mean of 70% (range 61–77%) correct implementation during baseline to a mean of 91% (range 82–96%) during BST training tests. P2 scored a mean of 97% (range 95–98%) across three extension tests and 97% correct implementation during the setting-extension test. P3 exhibited a mean of 39% (range 24–53%) correct implementation across five baseline tests, which increased to a mean of 92% (range 76–96%) during the BST training tests. P3 needed two additional BST sessions beyond the three initial extension tests to achieve generality of DTT skills. P3 demonstrated a mean of 94% (range 92–96%) correct implementation during the last three extension tests and 97% correct implementation of the DTT components during the setting-extension test. The mean percentage of correct DTT implementation across participants was 97% (range 94–99%) during post-tests. Finally, the mean percentage of correct DTT implementation across participants was 97% (range 94–99%) during maintenance tests, three weeks after the extension tests were conducted. Thus, BST training established and maintained generalized DTT skills across instructional programs and in a setting that differed from treatment.

The social validity survey results are provided in Table 1. Among the BST steps, feedback was most preferred (*M* = 5, *Mdn* = 3.67; question 1) and rehearsal (role-play) was the least preferred (*M* = 2.33, *Mdn* = 2). Collectively, participants rated feedback as the most effective BST step (*M* = 4.6, *Mdn* = 5; Question 2), instruction received the second highest rating (*M* = 4.33, *Mdn* = 4), while rehearsal (*M* = 3.33, *Mdn* = 4) and modeling (*M* = 2, *Mdn* = 2) were rated as less effective. This is consistent with component analyses of BST that have found feedback to be the most effective component (e.g., LaBrot et al., 2018). Individually, P1 rated rehearsal as the most effective step (Question 3), P2 rated instruction as the most effective, and P3 rated feedback as the most effective BST step. Regarding which BST step the participants liked the most (Question 4), P1 liked the instruction the most, while P2 and P3 liked the feedback the most. Finally, the participants reported experiencing the most improvement on effective reinforcement delivery (Question 5, *M* = 4.66, *Mdn* = 5), and they felt more competent (Question 6) when teaching the student (*M* = 4, *Mdn* = 4).

This study investigated the extension of correct implementation of DTT skills across diverse academic instructional programs by educators at an elementary school and, thus, expands on prior research in this area (e.g., Briggs et al., 2024; Hickey et al., 2024). The generality of DTT skills is important as school staff must exhibit novel behavior (not directly trained) to effectively teach a range of diverse skills to students (Sarokoff & Sturmey, 2008). DTT skills can be considered different members of a functional operant class in which the antecedent behavior from the staff varies across instructional programs or novel responses emerge within the class. In other words, the generality of DTT skills has occurred when staff can correctly implement DTT skills in the presence of different instructional targets that do not share any similar properties with the targets used during training.

In addition, our results support findings of previous studies that have evaluated BST as a socially valid training approach (e.g., Sarokoff & Sturmey, 2008). The experience of learning something perceived as useful (c.f. social validity) is a key finding, as participants experience may play a role in the maintenance of acquired DTT skills. Further studies could further examine the social validity of different BST steps. Finally, the present study supports previous research that has found that instruction alone has limited effectiveness (e.g., Clayton & Headley, 2019; Sarokoff & Sturmey, 2008) at establishing DTT skills. Baseline tests following vocal and written instructions yielded variable performance, and DTT skills did not improve until the remaining BST components were introduced.

A limitation of this study is that generality tests were not conducted during baseline. Thus, we cannot compare participant performance on generality tests prior to and after BST. Consequently, in the present experiment, we omitted the term generality. However, participants had little to no prior experience implementing DTT, and the five instructional programs used during extension tests were distinctly different from the instructional program (i.e., 10-friends) used during BST. Therefore, it is likely that the procedures used in this study represent a fair test of generality. Future studies should consider conducting generality tests during baseline to provide a stronger demonstration of generality.

A second limitation is that we did not measure the duration of BST training. This information can be important for school administrators to consider when seeking to adopt staff training procedures that are both time- and cost-effective. A final limitation is that the instruction step of BST was provided only before the first baseline test of DTT, consistent with Sarokoff and Sturmey (2008) but different from Ward-Horner and Sturmey (2008). Rather, we isolated the instructions step of BST during baseline to measure the effects of this step alone.

In this study, BST was used to teach DTT skills to three teaching assistants at a primary school. These DTT skills were extended across various teaching programs and were maintained for at least three weeks following BST. Additionally, the skills were extended to a setting that were not part of the original training. Notably, extension of DTT skills across instructional programs was observed in all three participants, although one participant required additional BST sessions to achieve the mastery criterion during the extension tests. Moreover, participants provided positive ratings of BST in a social validity survey following the intervention. The costs associated with delivering services to students with disabilities are high, and it is essential that those providing services are well-trained to make the most of available resources. BST can help make better use of available resources in primary schools by establishing a range of essential skills.

## Electronic supplementary material

Below is the link to the electronic supplementary material.

Supplementary file1 (DOCX 135 KB)

## Data Availability

The authors confirm that the data supporting the findings of the present study are available within the article and its supplementary materials. Anonymous raw score data can be obtained by contacting the authors.

## Appendix A

See Table 2

**Table 2:** An overview of the components measured during discrete trial teaching

(1) Discriminative stimulus (S^D^), for example a vocal instruction, must be clearly defined and easy distinguishable, with a distinct beginning and end
(2) The S^D^ should be appropriate for the task. For instance, if the participant was supposed to instructing the student to find the 10-friend of 3, they should avoid making mistake such as saying, “What is the 10-friend for 5”?
((3) S^D^ is the same within the same teaching target
(4) S^D^ is not interrupted
(5) When S^D^ is presented, the staff must time the delivery of antecedent to ensure that the student pay attention to the tasks or to the participants, indicated by turning his head towards the participant (i.e., exhibiting no “off-task” behavior) and the student demonstrates «ready position» by sitting up straight in the char with the hands on the table
(6) Prompts must be effective, producing the correct response
(7) Consequences must be delivered immediately after the correct response or within 3 s
(8) The consequences must be contingent, meaning they are presented only on correct responses (e.g., the S^R^s). An extinction condition was implemented when an incorrect response was emitted
(9) Consequences should be unambiguous (i.e., don’t saying “Wrong” with a smile or “Good job” with a furrowed brow)
(10) Consequences should be consistent. Reinforcement should follow each correct response. Incorrect responses or inappropriate behavior were followed by no consequences, or no attention were given

The components of Discrete Trial Teaching are based on Koegel and Rincover (1977). S^D^ = Discriminative stimulus and S^R^ = reinforcing stimulus.
