# Supplementary material for: Using Behavioral Skill Training to Extend Discrete-Trial Teaching Skills Across Instructional Programs
Source: Behav Anal Pract. 2025 Sep 25;19(3):1399–405. doi: 10.1007/s40617-025-01094-5 (PMC13638911; doi:10.1007/s40617-025-01094-5)
Supplement: Supplementary file 1 [file 40617_2025_1094_MOESM1_ESM.docx]

**Supplementary Materials**

**Example of a Scoring Sheet Containing 10 trials––Each Block Consisted of 10 Trials**

| **Trials** | Set a + (plus) for mastery and a – (minus) for incorrect response, prompted response or no response | **Trials** | Set a + (plus) for mastery and a – (minus) for incorrect response, prompted response or no response |
| --- | --- | --- | --- |
| **5+5** |  | **0+10** |  |
| **2+8** |  | **7+3** |  |
| **7+3** |  | **9+1** |  |
| **1+9** |  | **6+4** |  |
| **4+6** |  | **8+2** |  |
| **8+2** |  | **4+6** |  |
| **9+1** |  | **5+5** |  |
| **10+0** |  | **2+8** |  |
| **3+7** |  | **1+9** |  |
| **6+4** |  | **3+7** |  |

*Note.* The table shows to different examples of two blocks of Discrete-Trial Teaching according to a trial-by-trial-scoring sheet. The participants used different sheets in diverse teaching programs in which the targets were randomly rotated across teaching blocks.

**An Overview of Behavioral Skills Training**

| Instruction |  |
| --- | --- |
| (1) The experimenter handed out a written list of the ten components of DTT |  |
| (2) The experimenter handed out an operationalized definition of DTT orally |  |
| Baseline probes |  |
| (3) The experimenter sat a timer on 5 min |  |
| (4) During baseline probes the participants sat with the student and the experimenter presented the instruction «Do a teaching session in accordance with discrete trial teachin*g* with ten-friends in 5 min, do as best you can». Then, the participants conducted a 5 min session with DTT |  |
| BST-supervision |  |
| (5) The experimenter showed a short video clip that illustrated how a teaching session should be conducted, including the component of DTT. The participants watched the video clip at least three times |  |
| (6) The participants underwent a role play with the experimenter who played the role as the student, while the participants played the role as the teacher. During rehearsal the participants received supervision on how to use progressive delay prompts (Table 3), in addition to how to deliver vocal instructions effectively, response definitions (the student’s behavior) and how to deliver reinforcers effectively |  |
| (7) During the rehearsal, the experimenter provided oral feedback, which included behavior-specific praise and/or corrective feedback. Additionally, the experimenter offered feedback based on observations from the previous session. Corrective feedback was limited to one statement for every three positive feedback statements |  |
| (8) The experimenter sat a timer on 5 min and then, give the instruction «Do a teaching session in accordance with discrete *trial teaching* with 10-friends in 5 min, do as best you can». Next, the participants conducted a 5 min session with DTT |  |
| (9) Phase 5–8 was repeated until the participants obtained the mastery criterion of a minimum 90 % in three subsequent sessions |  |

*Note.* DTT = Discrete trial teaching.

**Illustration of the Task Material for Teaching Ten-Friends**

*Note.* The numbers in the top panel represent possible choices of 10-friends. The experimenter placed one of the numbers from the top panel in one of the boxes in the bottom panel and then presented the correct vocal discriminative stimulus.
